# The New York Institute of Stomatology

**Published:** 1904-07

**Authors:** 

**Affiliations:** The New York Institute of Stomatology


### Reports of Society Meetings.

### THE NEW YORK INSTITUTE OF STOMATOLOGY.

A MEETING of the Institute was held on Tuesday evening, March 1, 1904, at the "Chelsea," No. 222 West Twenty-third Street, New York, Dr. Brockway, the President, in the chair.

The minutes of the last meeting were read and approved.

Dr. G. C. Ainsworth, of Boston, read a paper entitled "Some Thoughts regarding Methods and a New Appliance for moving Dislocated Teeth into Position."

(For Dr. Ainsworth's paper, see page 481.)

Dr. Sinclair Tousey, A.M., M.D., read a paper at a meeting held at his residence, March 1, 1904, on the "Radiotherapy in Pyorrhœa Alveolaris, and Dental Radiography."

(For Dr. Tousey's paper, see page 495.)

Dr. E. A. Bogue stated that he had found Dr. Ainsworth's methods such a revelation to him that, so far as he had understood them, he had adopted them in several cases. Regarding the inclined plane shown by Dr. Ainsworth, Dr. Bogue thought if it would work as well for the rest of us as it did for Dr. Ainsworth it would assist very materially in obtaining a proper occlusion in certain cases. Dr. Bogue would also like to call particular attention to these little tubes that Dr. Ainsworth solders to the bands. These tubes accurately fit the spring-wire they are made to hold, so that there is no lost motion and the tooth is moved exactly as desired by properly bending the wire.

In considering the three appliances of Dr. Ainsworth, it seemed proper to call attention to a few collateral facts. The first was the contraction of the upper arch. He had noticed this in examining his own models, and his attention had also been called to it by other orthodontists. Dr. Stanley had confirmed these observations recently, asserting that seventy per cent. of the cases that needed regulating had contracted upper arches. If this were anything like the proportion, and Dr. Bogue believed it to be within the limits, there was a broad field for the use of Dr. Ainsworth's appliance among persons who could not give a great deal of time to having their teeth regulated. It was also a great saving of time and attention to those who were going to do the regulating. Without claiming that Dr. Ainsworth's method was applicable in all cases, it seemed to Dr. Bogue that it was a decided advance for a great many. Dr. Bogue would give an example or two. Among the lantern slides that he exhibited before this Institute a year ago was one of a little girl whom he had sent to a friend in Paris for treatment. This friend had applied to her teeth the Angle arches, above and below, making an admirable attachment and ligating the teeth so well that the fixtures, when Dr. Bogue first saw them, had been on about ten months. He had taken careful impressions and made models of the teeth as they then were. The gain in expansion of the entire arches had been very little. He could judge of this accurately, as he had the original models, and he measured them as they were originally and as they came to him about two years later. The little girl was impatient of pressure and intolerant of pain. He found that with a single nut at the end of each screw of the expansion arch those nuts could never be kept tight twenty-four hours. The moment the teeth responded to the immediate pressure the nuts became loose and turned backward. He sent this child to Dr. Ainsworth, who put on to the upper teeth such a fixture as he had described this evening. Dr. Bogue had replaced the Angle appliance on the lower teeth, but had taken the precaution to put two nuts on the screw at each end. By actual measurement, more progress was made in three weeks than had been made in the previous ten months, and this without pain. The patient was about thirteen years of age. Dr. Bogue had noticed recently that a new regulating appliance had been under discussion in the American Dental Club of Paris since this child had gone back.

Another case was that of a little girl seven years of age upon whom he had placed this fixture. He had seen her again after two days and again after two weeks. The gentleman into whose hands he had sent her reported that aside from drawing a developing central incisor slightly forward, he had had nothing to do but to keep the fixtures in place. He said the case might be regarded as practically completed; for, having moved the temporary molars and cuspids and the permanent central incisor out of the way of the lower teeth, the child had voluntarily assumed the proper occlusion; and the principal upper molars, which when he began had occluded one full cusp in advance of their proper position, now occluded with the anterior buccal cusp in the buccal depression of the lower molars, leaving plenty of room for the comfortable eruption of all the remaining teeth.

It was well to notice, as Dr. Ainsworth said, that the tooth may sometimes start suddenly and come out too far. Dr. Bogue had seen three cases where the expansion arch had pushed or pulled the teeth in a direction exactly opposite from that which was intended, because the gentleman into whose hands the cases were sent had failed to take cognizance of the fact that the arch is at work the entire twenty-four hours of the day, although the patient might not be aware that it was doing anything.

Dr. Bogue would take issue with Dr. Ainsworth when he stated that the lower teeth were inferior in stability to the upper ones. It seemed to him that the teeth in the lower jaw were far more stable than those in the upper jaw, and more difficult to move.

Dr. Ainsworth made an admirable use of his expansion arch in correcting deformities in the middle-aged. If we were not discussing his three regulating appliances only, he would feel like making his compliments to Dr. Ainsworth for the case of restoration shown, the patient being forty-nine years of age.

He would like to ask Dr. Ainsworth what solder he used in making these regulating appliances.

Dr. Bogue's attention had been attracted to this method, especially for use with little children, in which cases it had shown itself to be particularly well adapted to do the work required quickly and with little if any pain.

Dr. Bogue had been frequently asked why the permanent teeth should be so much larger than the temporary teeth they replace, and why these teeth should seem so out of proportion to the mouths in which we found them. He would like to ask a question,—Why are the feet of a pup so out of proportion to the size of the animal? Do we remove some of the toes lest the dog should seem to be all feet?

The retaining appliance shown commended itself, particularly for its cleanliness, acuracy, and comfort.

Dr. Rolof B. Stanley stated that the spirit which prompted him to take part in the discussion was born of a desire to learn rather than criticise. He had tried to analyze with an unbiassed mind Dr. Ainsworth's method of treatment of malocclusion and the application of the mechanical principle of his appliance. Dr. Stanley trusted he might be pardoned if for a moment he dwelt upon the A, B, C, of orthodontia, so familiar to all. It might serve to bring out more clearly the points he wished to make. Problems of orthodontia were problems of malocclusion, and restoration to the normal was the essential object. The first and most important step in any case was a correct diagnosis. The ability to make a correct diagnosis implied a knowledge of normal occlusion, of the fundamental types of malocclusion, and an appreciation of the harmony of facial lines. We must classify these cases and establish hard and fast rules, else the work savored of the experimental, or the operator must be guided by instinct alone. The only part individuality played in any case was in the degree of deviation from the normal. We could never bring order out of chaos if the individuality of the patient, as well as that of the operator, was allowed to play so important a part to the exclusion of fixed rules. With the knowledge of the normal occlusion and of the fundamental types of malocclusion, the deviation from the normal could be seen at a glance. It was the position of the teeth collectively that we must first consider, then the teeth separately, and an appliance should be chosen that would embody, in the order named, efficiency, simplicity, and inconspicuousness. Under the head of efficiency, the ideal appliance was one of standard pattern, no matter what the pattern might be, which would meet all the requirements from start to finish of all forms of malocclusion, and exclude the necessity of mechanical skill and ingenuity on the part of the operator. By simplicity was meant not only simplicity of mechanical construction, but freedom from an array of modifications or additions necessary to effect all changes in a given case, for the farther we wandered from the straight and narrow path of one fixed appliance the greater would be the confusion in the mind of the operator.

To consider the appliances shown, Dr. Stanley failed to see where they covered all the requirements, or how they were capable of accomplishing restoration to the normal in all cases. That they would bring about results in certain cases was proved by some of the photographs shown, but he doubted if they were available for all forms of malocclusion. For instance, where it was necessary to lengthen one or both lateral halves; where it was necessary to expand but one lateral half; where it was necessary to contract the lateral halves. Or, in the use of the inclined plane, how could the mesio-distal relation of the molars and bicuspids on one side only be restored; or in a case where the lower molars and bicuspids on one side are mesial or anterior and on the other distal or posterior to normal; how could the normal occlusion be restored by the use of the inclined plane or by any appliance or combination of appliances shown to-night? Dr. Stanley could cite many other cases that had come to his mind while listening to the paper, but thought these sufficient to emphasize the points he wished to make. They were all possible cases and cases that he had had in his practice during the present winter.

The question of discomfort to the patient and the length of time of treatment was of some moment. He believed in making orthodontic treatment as easy for both patient and operator as possible, both in application of the appliance to the teeth, the force used, and the time consumed, but he did not believe it to be of advantage to allow the patient to operate the appliance at will, since he did not appreciate the normal conditions. In conclusion, efficiency had been sacrificed in applying the principle of the expansion arch in the manner shown. While in some cases the ligature had been done away with to simplify the manipulation of the expansion arch, in as high as seventy per cent. of cases its use was required, either for carrying the incisors forward or for rotating them. Again this appliance presupposed that each tooth in the lateral halves was to be removed equally, which was far from the case generally. So with the inclined plane, the device might restore normal mesio-distal relation where both of the lateral halves required it, but could not where but one of the lateral halves required it. Cases of this type were as frequent as the bilateral. To meet all conditions, no matter how extreme, with one form of appliance certainly simplified treatment and promoted proficiency in its use.

Dr. J. Bond Littig was gratified to hear such a paper, and was going away with something new. He would especially commend the movable character of the appliance for retention. He thought there was great danger in an appliance impossible of removal. He had had two cases at least where, although thorough instructions had been given as to the proper cleansing of the immovable appliance, there had been a certain amount of disintegration of the enamel.

As to the inclined plane illustrated, Dr. Littig thought one principle was applied here that he had never seen before. We had all used the rubber plate, thickened in front to reduce the lower teeth or lengthen the molars, but he had never seen an appliance whereby the upper and lower teeth could at the same time be shortened.

Dr. C. F. Allan said that the paper had been most interesting to him. That effectiveness, simplicity, and cleanliness were salient points called for in all regulating and retaining appliances, and Dr. Ainsworth possibly more than anybody had attained these conditions. His only criticism would be the name of the paper. It seemed to him that the terms occlusion and malocclusion are much more properly descriptive of the conditions under consideration.

The inclined plane attached to the teeth of the upper jaw was the most individual of all the valuable appliances Dr. Ainsworth had shown, and Dr. Allan expected to put it in use immediately. Dr. Allan expressed his thanks to Dr. Ainsworth for the many good points he had given.

Dr. J. Morgan Howe, in expressing his gratification at the instructive paper, thought Dr. Ainsworth had presented an entirely new method of the application of springs. His own experience was that the movement of teeth by means of metal springs was the least painful of any of the various methods except the screw. The screw, however, requires more attention. Dr. Ainsworth's method certainly accomplished results in an easier and quicker way than those in general use. Whether or not it was universally applicable, it had added a new means of applying force and accomplishing results in certain cases at least. All of the presentations Dr. Ainsworth had made had been most interesting and instructive.

Dr. C. F. Allan stated that he wished to call attention to the necessity for always cementing on any appliance to be worn in the mouth and which cannot be removed for cleansing.

In putting on bands the teeth should be smeared with cement as well as the bands, and that this was imperatively necessary to prevent the possible rapid disintegration of the teeth over which appliances were placed, and in this matter one could not be too particular.

Dr. Bogue agreed with Dr. Allan regarding the cement under the bands of regulating and retaining appliances. He recalled one case at least from an exceedingly eminent orthodontist where, had the teeth been in his own mouth, he would rather have had them in any condition of dislocation than in the condition in which he found them. Dr. Bogue was not impressed with the fact that this appliance of Dr. Ainsworth could be removed to permit of a good appearance when the young lady went to a party. It was liable to form a habit that might be indulged in at other times.

Dr. Ainsworth, in closing the discussion, stated that he wished to disclaim all intention of developing a system of appliances to take the place of those now in use, or supplying them commercially; that he believed, as a rule, the appliance should be especially made for the case in hand. He had come here in response to the solicitations of members who were acquainted with what he was doing, to show what experience had dictated to him as improvements in the conduct of some cases. It had been claimed that seventy per cent. of all regulating cases required expansion of the arch. He thought this a sufficient apology for his demonstration of a self-acting appliance to do this work. Dr. Ainsworth had stated that this appliance was under the control of the patient by removing the front wire, but practically it was a control seldom exercised, as the appliance was so comfortable. It was very different from a rubber plate or an appliance that interfered with the functions of the mouth. It had been his experience that with such appliances the patient left them out much of the time.

These appliances were not perfect. The ideas were offered with the hope and belief that if intelligently applied they would be found to possess much merit. The principles might be applied in connection with other devices.

Dr. Charles O. Kimball, in proposing a vote of thanks to Dr. Ainsworth for coming on from Boston to give us this demonstration of his method, wished to call attention to two or three points; in the first place, to the simplicity, efficiency, and cleanliness of his apparatus, and no less so to the clear and simple and remarkably terse way in which he had demonstrated it by means of his photographs and his paper. Dr. Kimball would move a vote of thanks from the society to Dr. Ainsworth.

Motion seconded and carried.

Adjourned.

FRED. L. BOGUE, M.D., D.D.S.,
*Editor The New York Institute of Stomatology.*

